# Biopsy-Proven Pulmonary Tumors Detected by LDCT: A 10-Year Single-Center Study of Growth Patterns and Diagnostic Pitfalls

**DOI:** 10.7150/ijms.123625

**Published:** 2026-01-23

**Authors:** Chia-Tsung Hung, Chou-Chin Lan, Po-Chun Hsieh, Kun-Eng Lim, Tsung-Han Hsieh, I-Shiang Tzeng, Chih-Wei Wu

**Affiliations:** 1 Division of Thoracic Surgery, Department of Surgery, Taipei Tzu Chi Hospital, Buddhist Tzu Chi Medical Foundation, New Taipei City, Taiwan.; 2 Division of Pulmonary Medicine, Department of Internal Medicine, Taipei Tzu Chi Hospital, Buddhist Tzu Chi Medical Foundation, New Taipei City, Taiwan.; 3 Department of Chinese Medicine, Taipei Tzu Chi Hospital, Buddhist Tzu Chi Medical Foundation, New Taipei City, Taiwan.; 4 Department of Radiology, Taipei Tzu Chi Hospital, Buddhist Tzu Chi Medical Foundation, New Taipei City, Taiwan.; 5 Department of Research, Taipei Tzu Chi Hospital, Buddhist Tzu Chi Medical Foundation, New Taipei City, Taiwan.

**Keywords:** lung cancer screening, neoplasm progression, volume doubling time, image-guided biopsy, false-negative

## Abstract

**Introduction:**

Low-dose computed tomography (LDCT) reduces lung cancer mortality but may lead to increased resection of benign tumors. We aimed to characterize growth patterns, pathologic diagnoses, diagnostic timelines, and CT-guided biopsy accuracy in screening-detected lung tumors.

**Methods:**

We retrospectively analyzed LDCT screening data from 6,997 participants at Taipei Tzu Chi Hospital (2013-2018) with follow-up through 2023. Clinical, radiologic, and pathological features of biopsied lung tumors were evaluated. Volume doubling time (VDT) was calculated for progressive lesions.

**Results:**

Among the 128 patients who underwent biopsy, 84 were diagnosed with lung cancer, resulting in a detection rate of 1.2%. Of these, 86% were stage 0-I, and 95% were adenocarcinomas. Of the 157 biopsied tumors, 34% were benign. The most common benign pathological finding was fibrosis, followed by anthracosis. Lobulation and subsolid attenuation were significantly associated with malignancy. Time to diagnosis did not differ significantly between benign and malignant tumors (HR = 1.1, p = 0.53). Notably, 7% of benign tumors showed interval growth and 13% were detected de novo. Benign tumors exhibited a faster growth rate (3.8 vs. 1.5 mm/year, p = 0.28) and shorter VDT (347 vs. 565 days, p = 0.14) than malignant tumors. CT-guided biopsy had a 23% false-negative rate and a 78% negative predictive value.

**Conclusion:**

Benign tumors with interval growth or de novo presentation remain a diagnostic challenge. VDT alone is insufficient for distinguishing benign from malignant tumors. Given the substantial false-negative rate of CT-guided biopsy in small lung tumors, integrating radiologic features with clinical context is essential for guiding subsequent surveillance strategies.

## Introduction

Lung cancer is the leading cause of cancer-related mortality worldwide, accounting for approximately 1.8 million deaths annually 1. In Taiwan, lung cancer had the highest incidence rate among all malignancies in 2022, with 17,982 new cases (incidence rate: 77 per 100,000 population) and 10,053 deaths, according to the Ministry of Health and Welfare. Low-dose computed tomography (LDCT) is an effective screening tool for detecting asymptomatic pulmonary tumors at earlier stages and reducing mortality 2, 3. Its widespread use has increased the detection and treatment of early-stage lung cancer but has also led to increased surgical resection of benign tumors. Although various clinical and radiologic features associated with malignancy have been identified 4-8, the management of indeterminate pulmonary tumors detected by LDCT remains a major clinical challenge.

Growth kinetics, including changes in tumor diameter and volume doubling time (VDT), have been proposed as adjuncts for malignancy risk stratification. However, their clinical utility is limited by the marked variability in growth behavior among both benign and malignant lesions 9-12. A small subset of malignant tumors may even decrease in size during follow-up 11, and among growing tumors, growth patterns can range from linear to exponential 12. As most LDCT-detected tumors are small and peripheral, CT-guided lung biopsy is the preferred non-surgical diagnostic method; however, it is associated with substantial false-negative rates 13-15.

In this lung cancer screening study, we aimed to characterize the real-world diagnostic timelines and pathological findings of lung tumors confirmed through invasive procedures, compare clinical, radiologic, and growth characteristics between benign and malignant tumors, and evaluate the false-negative rate of CT-guided lung biopsy.

## Materials and Methods

We retrospectively reviewed the medical records of participants who underwent LDCT at Taipei Tzu Chi Hospital between January 1, 2013, and May 31, 2018, with follow-up until March 16, 2023. The analysis focused on lung tumors that were evaluated by invasive diagnostic procedures, and the pathological results of biopsied lesions were recorded. Some patients had multiple tumors requiring invasive procedures, and some tumors underwent more than one invasive procedure. Lung cancer staging was determined according to the American Joint Committee on Cancer (AJCC) TNM staging system, 9th edition.

In the real-world clinical practice setting of this study, no single standardized protocol existed for interpreting and reporting LDCT images. Nevertheless, our physicians adhered to two principal guidelines: Lung-RADS v1.0 to v1.1 16 and the 2017 Fleischner Society guidelines 4. The diagnostic pathway from initial LDCT screening to definitive invasive procedures was structured as follows: All initial LDCT examinations were interpreted by the institutional radiological staff. Radiologists systematically evaluated the images, documented findings, stratified malignancy risk, and recommended appropriate management strategies. For high-risk tumors, primary care physicians (predominantly family medicine specialists) in the health screening department conducted secondary image review and referred patients to subspecialty clinics in pulmonology or thoracic surgery. At the subspecialty level, pulmonologists or thoracic surgeons performed tertiary image assessment and re-evaluated malignancy risk stratification. They determined the optimal management approach: conservative surveillance with serial chest CT imaging, CT-guided biopsy, or direct surgical resection. The consulting specialists provided comprehensive counseling regarding the benefits and risks of invasive diagnostic interventions, ultimately arriving at a shared decision-making process with patients following extensive deliberation.

All radiologic images of biopsied tumors were independently reviewed by three experienced physicians: Chih-Wei Wu (pulmonologist, 16 years of thoracic radiology experience), Chia-Tsung Hung (thoracic surgeon, 10 years), and Kun-Eng Lim (radiologist, 34 years). Clinical characteristics collected included age, sex, comorbidities, smoking history, and family history of cancer. Radiologic features included positive chest X-ray (CXR) findings, CT attenuation, tumor size and morphology, and growth pattern during CT follow-up. CT attenuation was categorized as ground-glass opacity (GGO), part-solid, or solid; subsolid tumors were defined as those with GGO or part-solid appearance. Interobserver variability was assessed by tumor size measurement and CT attenuation classification (GGO, part-solid, or solid).

Tumor diameter was measured visually, and size was calculated as the average of the short and long axes. Growth patterns were classified into four categories: progressive, stationary, de novo, and without follow-up. Progressive tumors were defined as those with a ≥ 2 mm increase in diameter during follow-up and were used to calculate VDT. Stationary tumors showed an increase of < 2 mm and were excluded from the VDT calculation. De novo tumors were newly detected during follow-up. Tumors in the progressive, stationary, and de novo groups had at least one LDCT scan and one follow-up chest CT scan before undergoing invasive diagnostic procedures. The 'without follow-up' group included tumors with only one LDCT scan before invasive diagnostic procedures.

Real-time CT-guided biopsy images were retrospectively reviewed to assess visible pulmonary hemorrhage or pneumothorax on post-biopsy CT. Illustrative cases of tumor morphology are shown in the supplementary figures: lobulation (Supplementary Figure S1), air bronchogram (Supplementary Figure S2), spiculation (Supplementary Figure S3), bubble lucency (Supplementary Figure S4), and pleural tag (Supplementary Figure S5).

VDT was calculated using the modified Schwartz formula. Continuous variables were compared using the independent t-test or Mann-Whitney U test, and categorical variables using the chi-square or Fisher's exact test. Multivariate logistic regression was performed to identify risk factors for malignancy. The log-rank test was used to compare the time to diagnosis between malignant and benign tumors. We used the intraclass correlation coefficient (ICC) with a two-way random-effects model based on the average of 3 raters to assess tumor size variation across the three physicians. Fleiss' kappa was used to assess agreement in CT attenuation classification (GGO, part-solid, solid) among the three physicians. A p-value < 0.05 was considered statistically significant. Statistical analyses were conducted using GraphPad Prism version 10.3.1 and Python 3.14.0. The study was approved by the institutional review board with a waiver of informed consent (IRB approval number: 14-IRB073).

## Results

**Figure 1:**
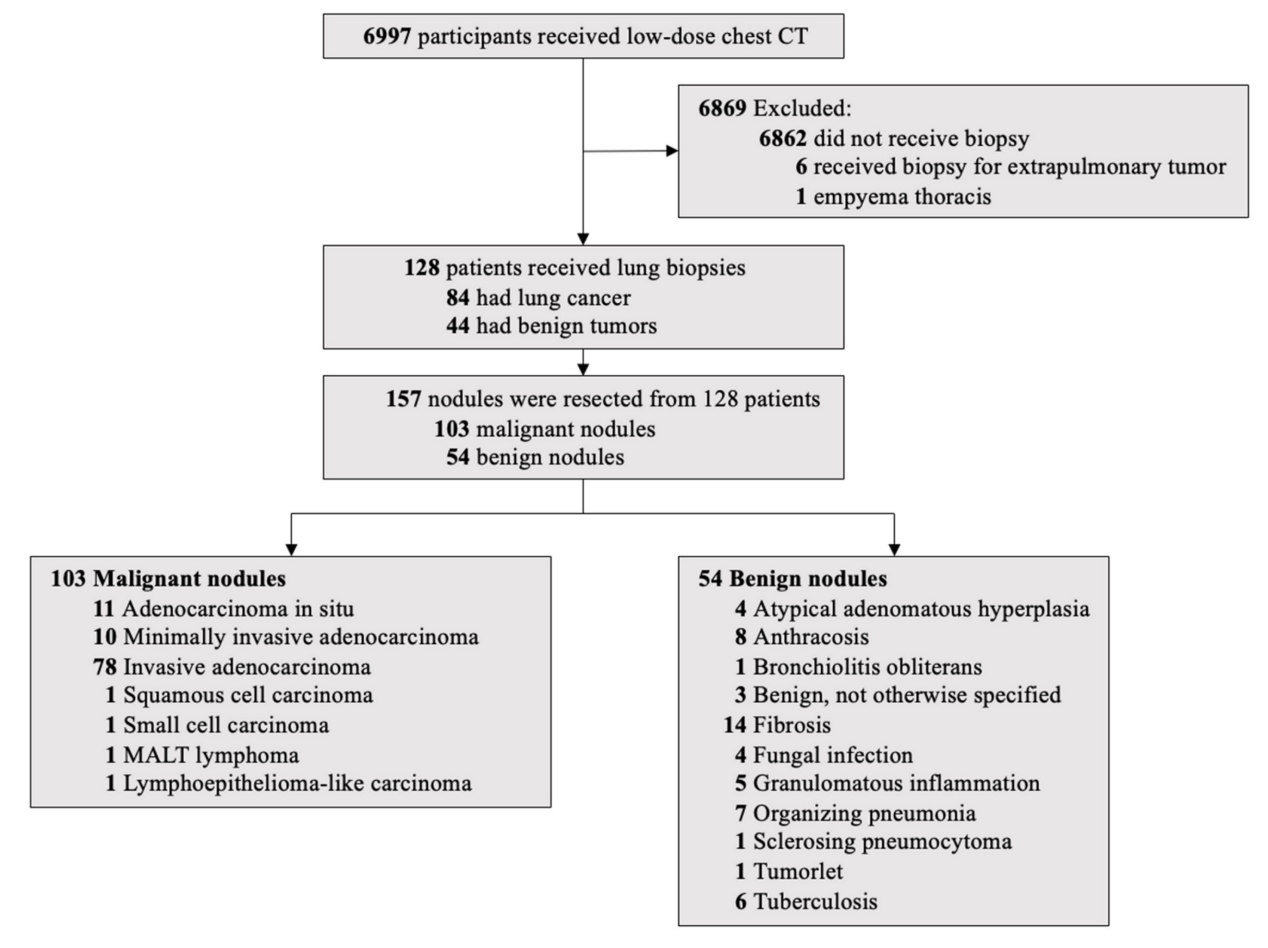
Flowchart of Lung Cancer Screening.

As shown in Figure 1, 6,997 participants underwent LDCT screening. After excluding 6,862 participants without biopsy, six who underwent biopsy for extrapulmonary tumors, and one with empyema thoracis, 128 patients underwent lung biopsy. Of these, 84 (66%) were diagnosed with lung cancer and 44 (34%) with benign lesions, yielding an overall lung cancer detection rate of 1.2% (84/6,997). A total of 157 tumors were biopsied from the 128 patients. At the tumor level, 157 tumors were confirmed pathologically: 103 malignant (66%) and 54 benign (34%).

### Pathologic diagnoses (Figure 1)

Among malignant tumors, invasive adenocarcinoma was the most common (n = 78), followed by adenocarcinoma in situ (n = 11), minimally invasive adenocarcinoma (n = 10), and one case each of squamous cell carcinoma, small cell carcinoma, mucosa-associated lymphoid tissue lymphoma, and lymphoepithelioma-like carcinoma. Benign tumors included fibrosis (n = 14), anthracosis (n = 8), organizing pneumonia (n = 7), tuberculosis (n = 6), granulomatous inflammation (n = 5), atypical adenomatous hyperplasia (n = 4), fungal infection (n = 4), benign tumor not otherwise specified (n = 3), and single cases of sclerosing pneumocytoma, tumorlet, and bronchiolitis obliterans.

### Clinical and radiologic features

Baseline characteristics are summarized in Table 1. Patients with malignant tumors were older than those with benign tumors (median 63 vs. 59 years; p = 0.039). No significant differences were observed in sex, smoking history, or family history of cancer. Diabetes mellitus was more frequent in malignant cases (22% vs. 7%; p = 0.019). Malignant tumors were undetectable on chest X-ray in 82% (84/103) of cases.

Radiologically, malignant tumors were larger (median 12 mm vs. 9.5 mm; p = 0.040) and more often presented as GGO (p = 0.001), part-solid (p = 0.025), or with lobulated margins (p = 0.004). Other CT features, including air bronchogram, spiculation, bubble lucency, and pleural tags, were not significantly different between groups. De novo tumors were more frequent among benign lesions (p = 0.033); other growth patterns showed no group differences. For interobserver variability, the ICC for tumor size was 0.98, and the Fleiss' kappa for CT attenuation classification was 0.81.

### Predictors of malignancy

Baseline characteristics that showed statistical significance in the univariate logistic regression analysis (Table 1)—including age, diabetes mellitus, size, subsolid attenuation, lobulation, and de novo presentation—were subsequently incorporated into the multivariate logistic regression model. Multivariate logistic regression (Table 2) identified subsolid appearance as the strongest independent predictor of malignancy (adjusted odds ratio [aOR] 7.19; 95% CI: 3.14-17.62; p < 0.001), followed by lobulation (aOR 4.88; 95% CI: 1.67-17.09; p = 0.007). Tumor size approached statistical significance (aOR 1.056; p = 0.055). De novo presentation was inversely associated with malignancy (aOR 0.197; p = 0.072).

### Stages and histologic subtypes

Of the 84 lung cancer patients, 80 (95%) had adenocarcinoma and 72 (86%) were stage 0-I (Table 3). Among the 99 adenocarcinoma tumors (including multiple lesions per patient), the lepidic subtype was most common (n = 42), followed by acinar (n = 15), papillary (n = 3), micropapillary (n = 2), and solid (n = 1); 36 were unclassified (Supplementary Table 1). EGFR mutations (Supplementary Table 2) included L858R (n = 33), exon 19 deletion (n = 11), and two cases with dual mutations.

### Diagnostic intervals and growth dynamics

**Figure 2:**
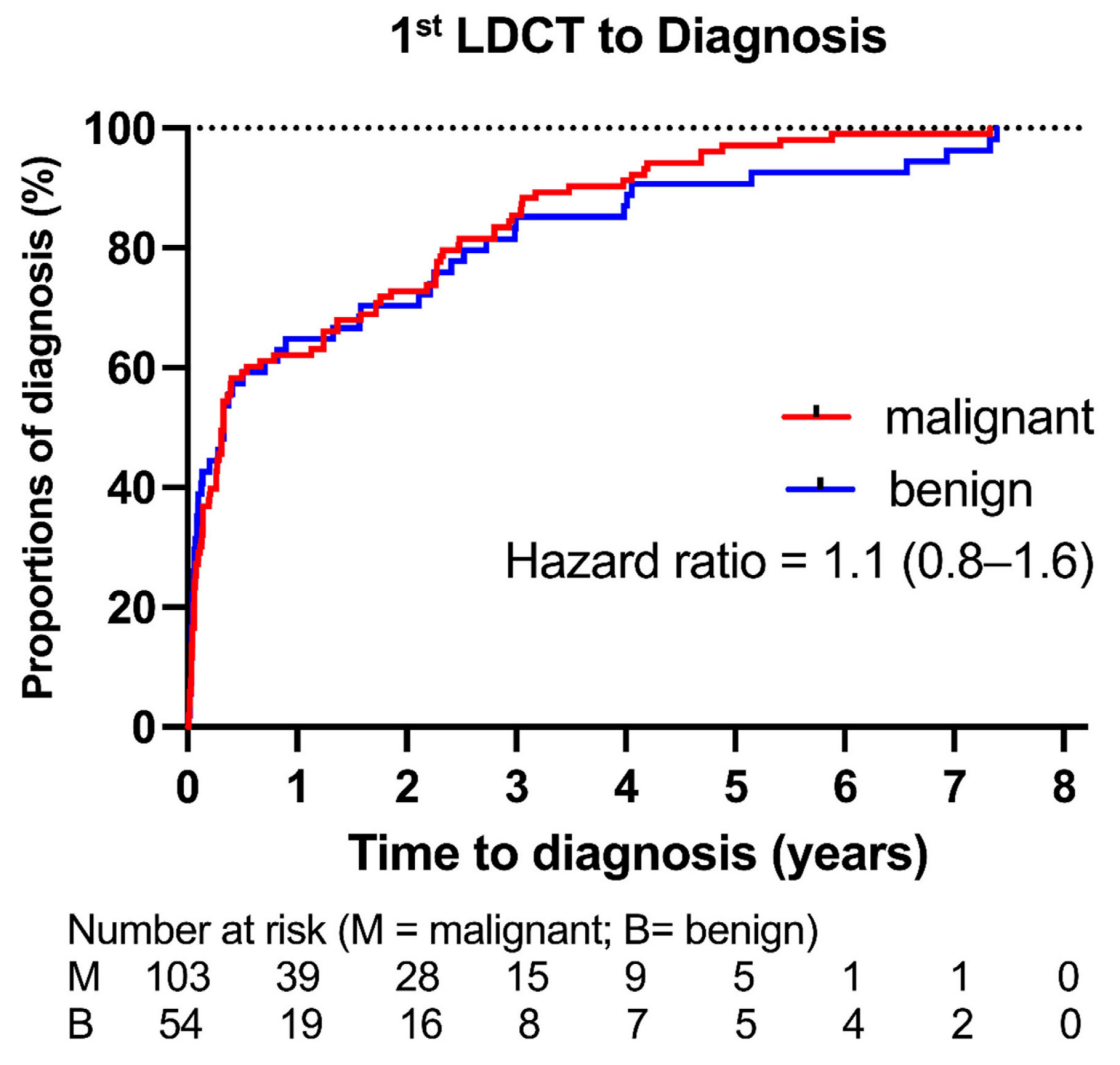
** Time from First LDCT to Final Pathological Diagnosis of Lung Tumors.** We used Kaplan-Meier analysis to illustrate the time to final pathological diagnosis for both malignant and benign tumors. There was no statistically significant difference between the two groups (hazard ratio [95% confidence interval], 1.1 [0.8-1.6]; P = 0.53).

Kaplan-Meier analysis (Figure 2) showed no significant difference in time to diagnosis between malignant and benign tumors (HR 1.1; 95% CI: 0.8-1.6; p = 0.53). Time from first LDCT to diagnosis varied by growth pattern (Supplementary Table 3): “without follow-up” tumors had the shortest interval (median 22 days), and de novo tumors the longest (median 1,667 days).

**Figure 3:**
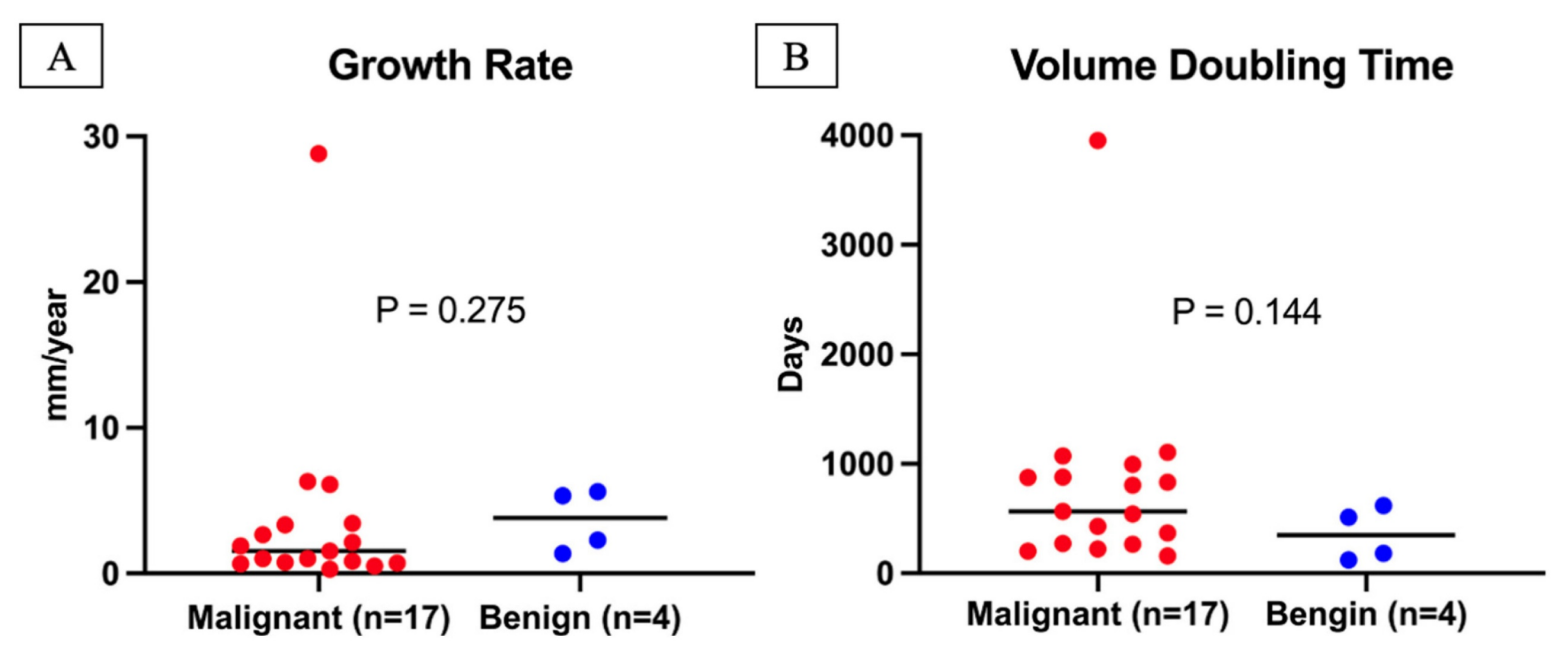
** Growth Rate and Volume Doubling Time of Progressive Lung Tumors.** A total of 21 tumors had an increase in diameter ≥2 mm during follow-up, consisting of 17 malignant and 4 benign tumors. (A) The growth rate of malignant tumors (median with range) was 1.5 mm/year (0.3-28.8 mm/year), while the growth rate of benign tumors (median with range) was 3.8 mm/year (1.4-5.6 mm/year). Growth rates did not differ significantly between malignant and benign tumors (P = 0.275). (B) The volume doubling time of malignant tumors (median with range) was 565 days (159-3,951 days), while that of benign tumors (median with range) was 347 days (120-618 days). Volume doubling times did not differ significantly between malignant and benign tumors (P = 0.144).

Among 21 progressive tumors (with ≥ 2 mm increase in diameter), benign tumors had a higher median growth rate (3.8 vs. 1.5 mm/year) and shorter VDT (347 vs. 565 days) than malignant tumors; however, these differences were not significant (p = 0.275 and p = 0.144, respectively) (Figure 3). Supplementary Figure 6 shows individual growth trajectories of the 21 progressive tumors: malignant (n = 17) in Panel A and benign (n = 4) in Panel B.

### CT-guided biopsy performance and complications

**Figure 4:**
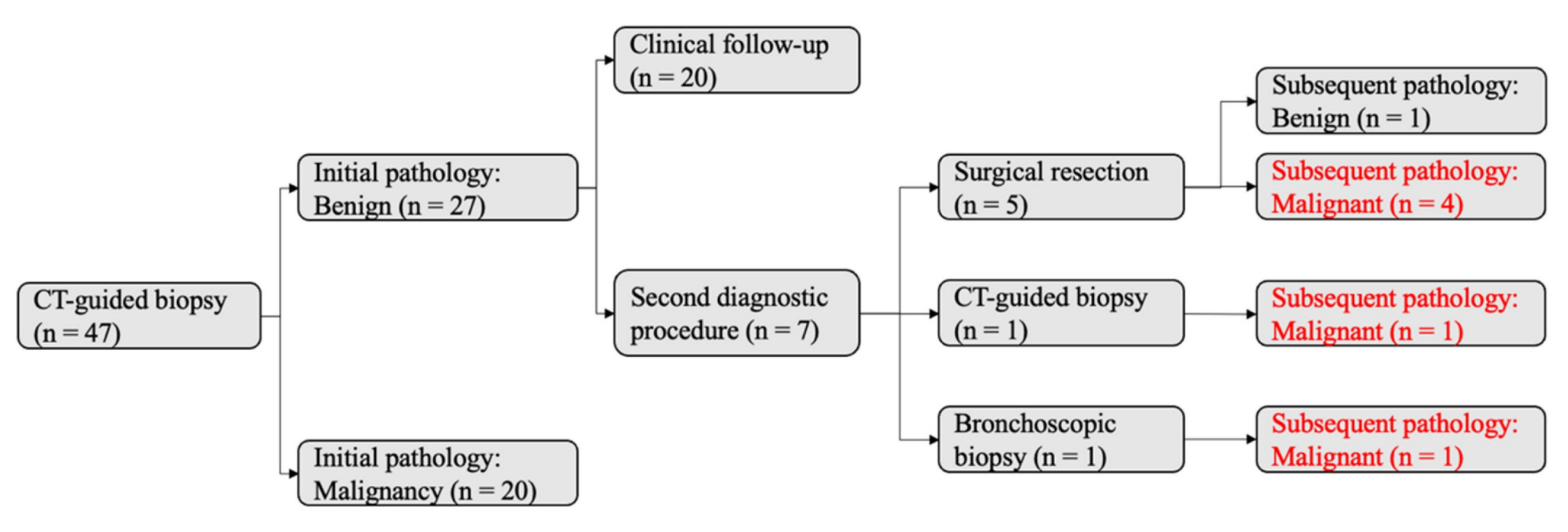
** Diagnostic Accuracy of Initial CT-Guided Biopsy.** A total of 47 tumors underwent CT-guided biopsy as the initial diagnostic procedure. The initial pathology reports identified 27 benign and 20 malignant tumors. Among the 27 tumors initially classified as benign, seven underwent a second diagnostic procedure based on clinical suspicion: five received surgical resection, one received an additional CT-guided biopsy, and one received bronchoscopic biopsy. Subsequent pathology revealed six malignant and one benign tumor. Notably, six of the 27 tumors initially classified as benign were later confirmed to be malignant during follow-up.

Of 47 tumors initially evaluated by CT-guided biopsy, 27 were diagnosed as benign and 20 as malignant (Figure 4, left). Among the 27 tumors in the initial benign group, 7 underwent additional diagnostic procedures, revealing 6 malignancies and 1 benign lesion. This corresponded to a false-negative rate of 23% (6/26) and a negative predictive value (NPV) of 78% (21/27). The reasons for the 7 additional procedures (Figure 4, right) were as follows: 3 tumors showed an increase in size during chest CT follow-up and thus received second invasive procedures (lobectomy, n = 1; CT-guided biopsy, n = 1; bronchoscopic biopsy, n = 1); 1 tumor had an adjacent new spiculated tumor in the same lobe during follow-up chest CT, thus receiving wedge resection of the two tumors; 1 tumor had an initial pathologic report of atypia with high suspicion for early lung cancer, and thus received direct lobectomy; 1 tumor had highly suspicious radiologic morphology (21-mm part-solid tumor with lobulation) despite an initial pathology report of atypical adenomatous hyperplasia, thus receiving direct lobectomy; 1 tumor received direct wedge resection because the patient desired certainty after discussion with the chest surgeon.

In total, 48 CT-guided biopsy procedures (Supplementary Table 4) were performed, with pulmonary hemorrhage in 75%, pneumothorax in 56%, and both in 42%; no cases required invasive intervention.

## Discussion

Our study found a lung cancer detection rate of 1.2%, with 86% at stage 0-I. Following the introduction of LDCT in Taiwan, two retrospective studies from southern Taiwan also reported increased early-stage (stage 0-I) diagnoses, while stage IV cases remained stable 17, 18. The largest prospective study in Taiwan, TALENT, screened 12,000 never-smokers or light smokers (50% with a family history of lung cancer) and detected lung cancer in 2.6% of participants at 1 year, with 19% being stage 0 cases 19. The higher detection rate in TALENT may reflect a greater prevalence of family history and a higher proportion of stage 0 cases (19% vs. 7% in our study). Surgeons in the prospective TALENT study may have adopted a more aggressive approach to resecting small lung cancers, leading to a higher resection rate for stage 0 disease.

According to Lung-RADS v2022 7, over 60% of screened individuals had lung tumors, creating a substantial follow-up workload. In our cohort, 34% of patients undergoing invasive procedures had benign lesions. While some of these resections identified treatable conditions such as tuberculosis or fungal infections, the most common diagnosis was fibrosis, consistent with previous reports 20, 21. A multicenter U.S. lung cancer screening study involving 9,050 participants reported that 15% (38/260) of surgical resections revealed benign pathology, with fibrosis accounting for 32% (12/38) of cases 20. Similarly, a single-center U.S. study of 4,798 participants found benign pathology in 13% (19/148) of resections, with fibrosis again being the most common finding (42%, 8/19) 21. Recognizing common benign pathologies and their imaging features may help reduce unnecessary surgical interventions.

Growth analysis revealed that 7% of benign tumors enlarged and 13% appeared de novo. The literature indicates that up to 50-77% of resected benign tumors in lung cancer screening programs may grow over time 20, 21. However, growth definitions vary; we applied a ≥ 2 mm increase in diameter, aligning with Fleischner Society recommendations 4. On the other hand, most de novo tumors in screening cohorts are benign. A study from England reported that approximately 10% of participants in a lung cancer screening program had newly detected lung tumors during follow-up; after clinical evaluation, 96% of these newly detected tumors were considered benign based on pathological diagnosis, radiologic features, or absence of cancer diagnosis in the national cancer registry 22. Alam et al. reported that 24% of resected benign lung tumors were newly detected lesions 20. Further studies are needed to elucidate the behavior of growing or de novo lung tumors and to reduce the risk of overdiagnosis.

In our study, benign tumors exhibited higher diameter growth rates and shorter VDTs than malignant tumors, although these differences were not statistically significant. This finding aligns with prior reports demonstrating substantial overlap in growth kinetics between benign and malignant lesions 10, 11. In a U.S. lung cancer screening cohort, Hammer et al. reported shorter VDTs for malignant tumors than for benign tumors (p = 0.2) 10. In contrast, Zhang et al. found the opposite trend in a routine clinical practice cohort in China (p = 0.18) 11. Both studies 10, 11, consistent with the NELSON trial 3, defined significant growth as a ≥ 25% increase in volume. In contrast, our analysis calculated VDT only for progressive tumors with a ≥ 2 mm increase in diameter. Emerging evidence suggests that VDT alone offers limited discriminatory value for growing tumors, underscoring the need to incorporate additional radiologic features into risk assessment.

In our screening cohort, the first-attempt CT-guided biopsy had an NPV of 78% and a false-negative rate of 23%. Screening-detected tumors are typically smaller than those found in routine practice, which increases nondiagnostic rates 23. Most biopsy accuracy studies are based on routine clinical populations 13-15. In a Chinese cohort of 861 patients with initial benign pathology and larger tumors (mean diameter 44 mm), the NPV was 84% 13; a French multicenter study of 149 negative biopsies reported an NPV of 51% 14; and a Korean multicenter study found an NPV of 60% among 2,590 nondiagnostic biopsies 15. False negatives remain common, with risk factors including limited radiologist experience, procedural complications, and the presence of atypical cells in initial specimens 13-15. Suspicious tumors with negative biopsy results require close follow-up and, if needed, repeat biopsy.

This study may underestimate the false-negative rate and overestimate the negative predictive value of CT-guided biopsy. A total of 47 tumors underwent CT-guided biopsy as the initial diagnostic procedure. Among the 27 tumors initially classified as benign, only seven underwent a second diagnostic procedure. The remaining 20 tumors underwent conservative chest CT surveillance because their morphology on imaging remained stable during the study period. However, these 20 tumors may represent early-stage lung cancer, such as adenocarcinoma in situ or minimally invasive adenocarcinoma. Thus, if the follow-up period were sufficiently long, these 20 tumors might exhibit significant morphological changes and warrant repeat biopsy. Consequently, the false-negative rate of CT-guided biopsy in this study may be underestimated, and the negative predictive value may be overestimated. Further long-term studies are needed, optimally with follow-up exceeding 10 years, to elucidate the indolent natural course of early-stage lung cancer.

Given the challenges in distinguishing benign from malignant tumors and the potential underestimation of false-negative rates in CT-guided biopsy, we propose several actionable strategies for screening programs and clinical practice. At the initial screening LDCT stage, radiologists should follow Lung-RADS v2022 7 to analyze the images and refer patients with category 3 or 4 tumors to a pulmonologist or chest surgeon for further management. In the outpatient setting, pulmonologist or chest surgeon should further incorporate the 2013 Brock score 6 to calculate individual cancer probability for each tumor. The newer 2017 PanCan score 8 may also be used as a complementary tool. For tumors that remain indeterminate even after biopsy, the case should be discussed at a multidisciplinary tumor board, which comprises radiologists, pulmonologists, chest surgeons, medical oncologists, nuclear medicine physicians, and pathologists, to reach a consensus on further management. In addition, positron emission tomography/computed tomography (PET/CT) or deep learning methods should be considered as part of the diagnostic workup and clinical decision-making process. The development of promising artificial intelligence methods 24 with diagnostic performance comparable to human physicians has shown great potential. When incorporating these guidelines 4-8, radiologic features such as tumor lobulation (as observed in our study) or usual interstitial pneumonia should also be considered as predictors of malignancy.

In summary, most detected lung cancers in our screening program were early-stage, but benign lesions—some with growth or de novo presentation—remained a significant diagnostic challenge. Overlap in growth kinetics between benign and malignant tumors diminishes the utility of VDT alone. The limited accuracy of CT-guided biopsy further underscores the need for integrated diagnostic strategies. Combining radiologic features, clinical context, and longitudinal imaging may help minimize unnecessary interventions while preserving the benefits of early detection.

## Supplementary Material

Supplementary figures and tables.

## Figures and Tables

**Table 1:** Baseline Characteristics of Malignant and Benign Pulmonary Tumors (n = 157)

Characteristics	All (n = 157)	Malignant (n = 103)	Benign (n = 54)	P-value
Age, median (range), years	62 (36-84)	63 (36-84)	59 (41-84)	0.039
Sex, No.				0.497
Male	64	40	24	
Female	93	63	30	
Comorbidities, No.				
Emphysema	8	3	5	0.125
Past cancer history	7	4	3	0.692
Diabetes mellitus	27	23	4	0.019
Hypertension	41	28	13	0.673
Chronic hepatitis	10	7	3	0.999
Smoking history, No.				0.324
Yes	31	18	13	
No	126	85	41	
Family cancer history, No.				
Lung cancer	11	7	4	0.999
Extrapulmonary cancer	15	7	8	0.104
Location, No.				0.398
Upper lobe	77	48	29	
Non-upper lobe	80	55	25	
Size^a^, median (range), mm	11 (2-48)	12 (3-44)	9.5 (2-48)	0.040
Chest X-ray findings, No.				0.572
Positive	31	19	12	
Negative	126	84	42	
CT attenuation, No.				
Ground-glass opacity	59	48	11	0.001
Part-solid	27	23	4	0.025
Solid	71	32	39	< 0.001
Tumor morphology^b^, No.				
Lobulation	39	33	6	0.004
Air bronchogram	25	16	9	0.854
Spiculation	23	13	10	0.321
Bubble lucency	20	15	5	0.344
Pleural tag	50	31	19	0.516
Growth patterns, No.				
Progressive^c^	21	17	4	0.141
Stationary^d^	58	39	19	0.741
*De novo* ^e^	10	3	7	0.033
Without follow-up^f^	68	44	24	0.836

**Abbreviations**: CXR, Chest X-ray; GGO,ground-glass opacity. Footnotes: ^a^ Tumor size was recorded at the time of initial detection. ^b^ Tumor morphological features may overlap. ^c^ Progressive tumors: increase in diameter ≥ 2 mm during follow-up. ^d^ Stationary tumors: increase in diameter < 2 mm during follow-up. ^e^ *De novo*: Newly detected tumors during follow-up. ^f^ Tumors that had only one LDCT scan before undergoing invasive diagnostic procedures. Note: The tumors in the progressive, stationary, and se novo groups had at least one LDCT and at least one follow-up chest CT scan before undergoing invasive diagnostic procedures.

**Table 2:** Multivariate Logistic Regression Analysis of Risk Factors Associated with Malignancy

Variables	Adjusted Odds Ratio (95% CI)	P-value
Age	1.026 (0.979-1.077)	0.286
Diabetes mellitus	2.763 (0.848-11.04)	0.113
Size	1.056 (1.001-1.121)	0.055
Subsolid	7.195 (3.138-17.62)	< 0.001
Lobulation	4.878 (1.674-17.09)	0.007
*De novo*	0.197 (0.028-1.034)	0.072

**Abbreviations**: CI, confidence interval; Subsolid = ground-glass opacity + part-solid tumor.

**Table 3:** Histological Subtypes and Clinical Stages of Lung Cancer (n = 84)

Stages	Adenocarcinoma(n = 80)	Squamous Cell Carcinoma (n = 1)	Small Cell Carcinoma (n = 1)	MALT Lymphoma (n = 1)	Lymphoepithelioma-like Carcinoma (n = 1)
0	6	0	0	0	0
I	64	1	0	0	1
II	2	0	0	0	0
III	6	0	0	1	0
IV	2	0	1	0	0

**Abbreviation**: MALT lymphoma, mucosa-associated lymphoid tissue lymphoma.

## Abbreviations

LDCT: low-dose computed tomography
VDT: volume doubling time
AJCC: American Joint Committee on Cancer
CXR: chest X-ray
GGO: ground-glass opacity
ICC: intraclass correlation coefficient
aOR: adjusted odds ratio
NPV: negative predictive value
