# Supplementary material for: Biopsy-Proven Pulmonary Tumors Detected by LDCT: A 10-Year Single-Center Study of Growth Patterns and Diagnostic Pitfalls
Source: Int J Med Sci. 2026 Jan 23;23(3):780–7. doi: 10.7150/ijms.123625 (PMC12964566; doi:10.7150/ijms.123625)
Supplement: Supplementary file 1 — Supplementary figures and tables. [file ijmsv23p0780s1.pdf]

Supplementary materials:

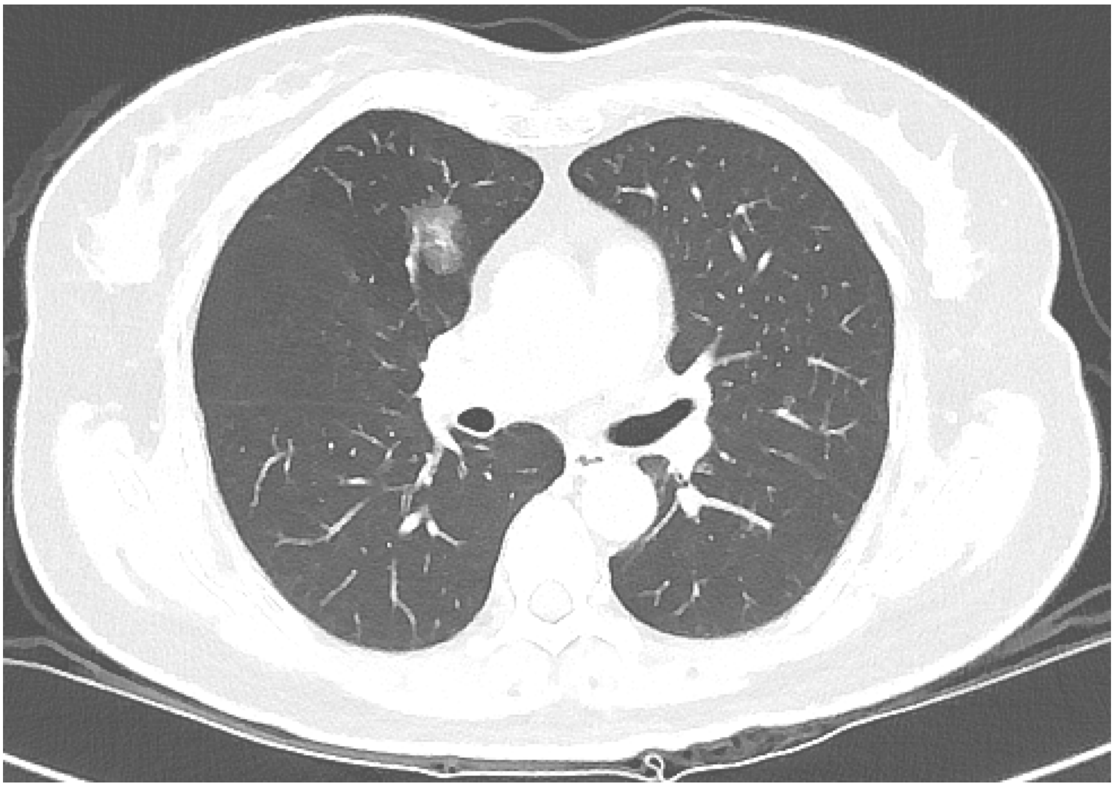

Supplementary Figure 1. Tumor with Lobulation

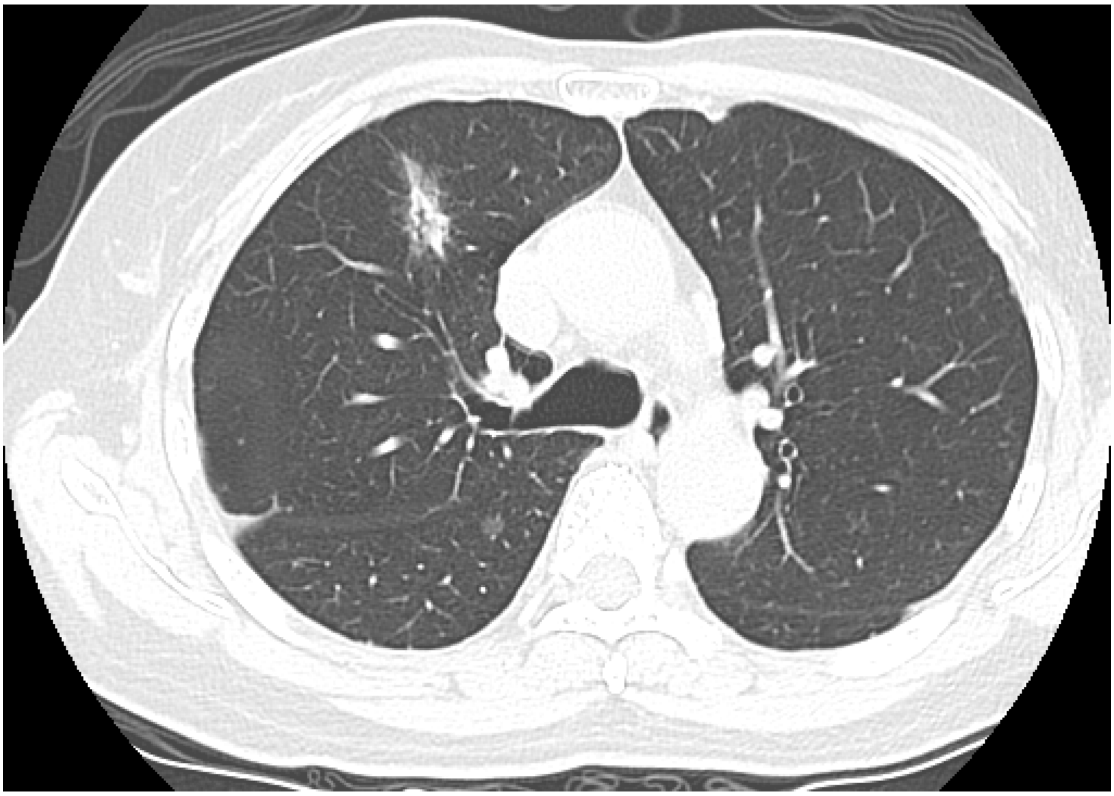

Supplementary Figure 2. Tumor with Air Bronchogram

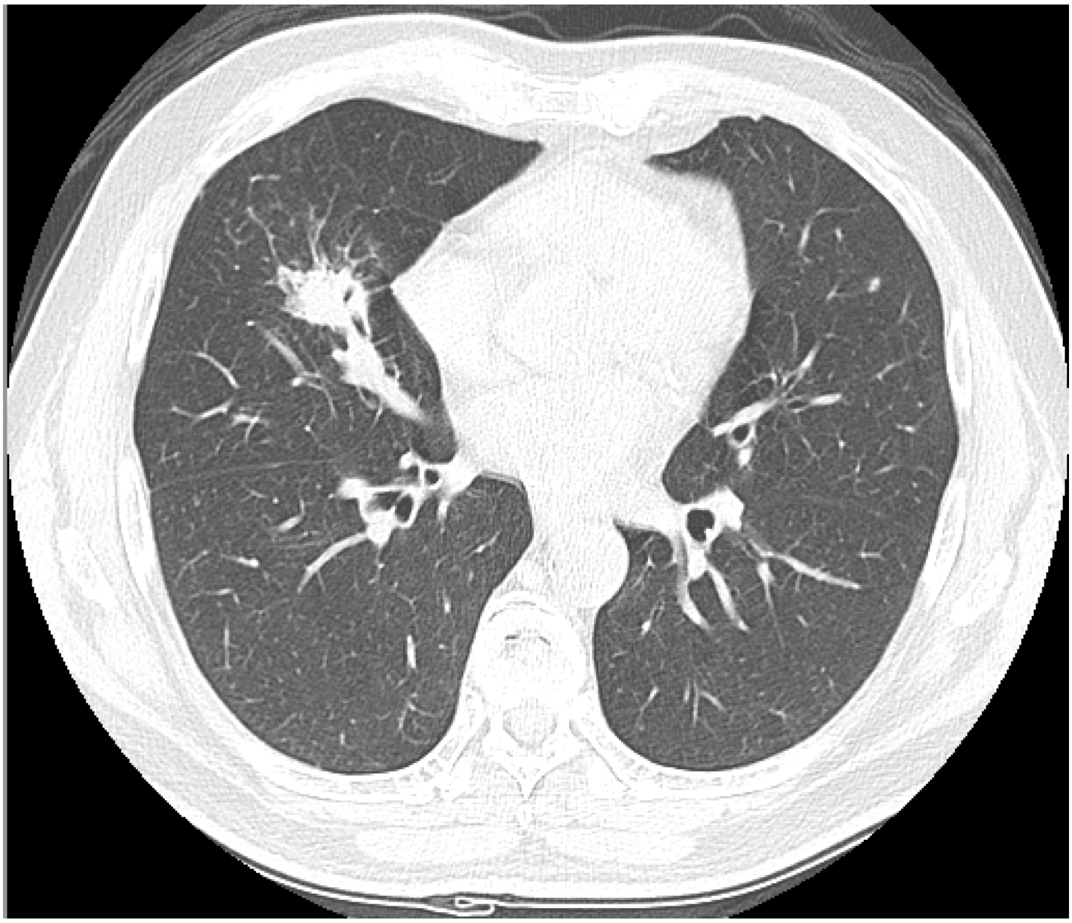

Supplementary Figure 3. Tumor with Spiculation

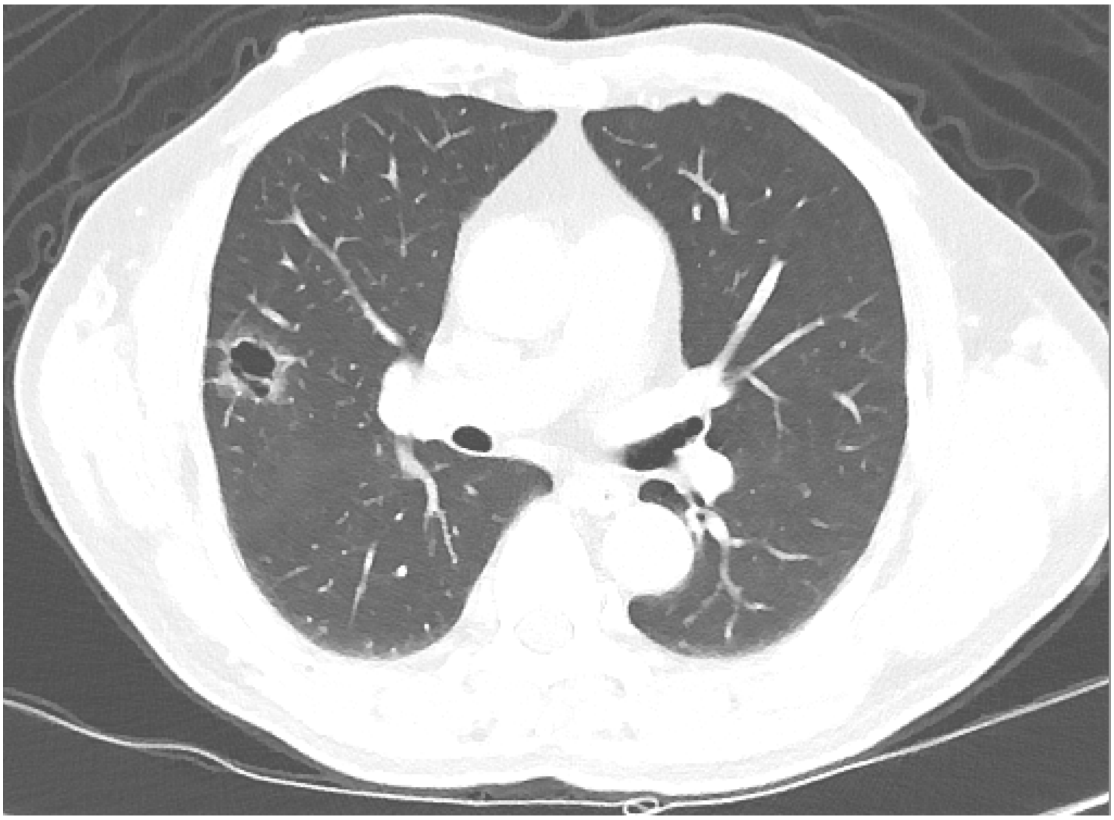

Supplementary Figure 4. Tumor with Bubble Lucency

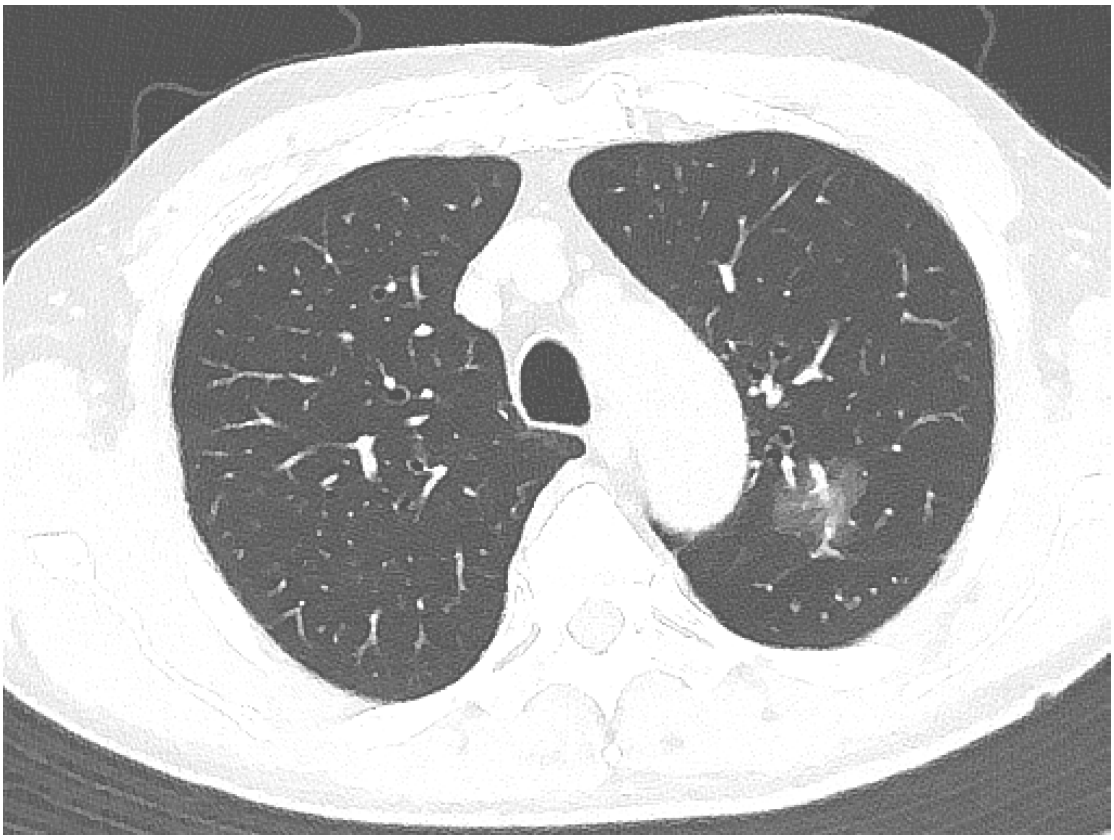

Supplementary Figure 5. Tumor with Pleural Tag

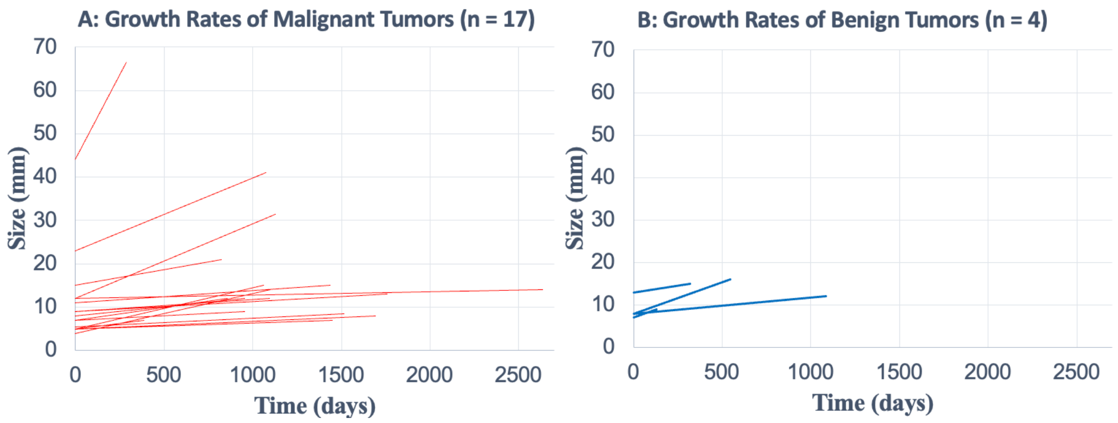

Supplementary Figure 6. Growth Rates of Lung Tumors

A total of 21 tumors showed an increase in size during follow-up. Panel A illustrates the growth rates of malignant tumors (n = 17), while Panel B depicts the growth rates of benign tumors (n = 4).

Supplementary Table 1. Histological Subtypes of Adenocarcinoma (n = 99)

**Histologic Subtypes of Adenocarcinoma**	**Lepidic**	**Acinar**	**Papillary**	**Micropapillary**	**Solid**	**Unknown**
Total number of tumors = 99	n = 42	n = 15	n = 3	n = 2	n = 1	n = 36

Note: The 99 adenocarcinoma tumors included multiple tumors from some patients.

Supplementary Table 2. EGFR Mutation Subtypes in Adenocarcinoma (n = 99)

EGFR Subtypes	Exon19	Exon20	G719X	L858R	S768I	T790M	Not Detected	Unknown
Total number of tumors = 99	n = 11	n = 5	n = 1	n= 33	n = 1	n = 1	n = 18	n = 31

Abbreviation: EGFR, epidermal growth factor receptor mutation.

Note: The 99 adenocarcinoma tumors included multiple tumors from some patients. Two tumors had multiple EGFR mutations: one with L858R and S768I, and another with an exon 19 deletion and T790M.

Supplementary Table 3. Time from the First LDCT to Final Diagnostic Procedure Stratified by Tumor Growth Pattern

**Tumor Growth Pattern**	**n = 157**	**Time to First Diagnostic Procedure, Median (Range), Days**
Progressive	n = 21	1,080 (149–2,674)
Stationary	n = 58	452 (97–2,674)
De novo	n = 10	1,667 (831–2,698)
Without follow-up	n = 68	22 (3–101)

Definitions:

• Progressive tumors: increase in diameter ≥ 2 mm during follow-up.

• Stationary tumors: increase in diameter < 2 mm during follow-up.

• De novo: newly detected tumors during follow-up.

• Without follow-up: Tumors that had only one LDCT scan before undergoing invasive diagnostic procedures.

Note: The tumors in the progressive, stationary, and de novo groups had at least one LDCT and at least one follow-up chest CT scan before undergoing invasive diagnostic procedures.

Supplementary Table 4. Complications of CT-Guided Biopsy (n = 48 Procedures)

**CT-guided Biopsy Complications**[a]	**Pulmonary Hemorrhage**	**Pneumothorax**	**Both**[b]
Total number of procedures = 48	n= 36	n = 27	n = 20

Footnotes:

[a] No invasive management was required for these complications.

[b] Both pulmonary hemorrhage and pneumothorax occurred in 20 procedures.
